# Microbiota–innate immune crosstalk drives atherosclerosis: mechanisms, disease progression, and emerging therapeutic strategies

**DOI:** 10.3389/fimmu.2026.1900899

**Published:** 2026-07-17

**Authors:** Yongang Li, Jinheng Zhu, Mingkui Huang, Xuan Liu, Liezhi Wang

**Affiliations:** 1 The First People’s Hospital of Wenling, Taizhou, China; 2 Wenzhou Medical College, Wenzhou, China; 3 Taizhou Central Hospital, Taizhou, China

**Keywords:** atherosclerosis, gut microbiota, inflammation, innate immunity, microbiota–immune crosstalk, precision therapy

## Abstract

Atherosclerosis (AS) is a complex cardiovascular disease driven by the interplay of dysregulated lipid metabolism, chronic inflammation, and immune dysfunction. Increasing evidence has revealed that the gut microbiota not only regulates host metabolic homeostasis but also actively contributes to the initiation and progression of AS through intricate interactions with the innate immune system. Microbial-derived signaling molecules, including lipopolysaccharides, outer membrane vesicles, extracellular nucleic acids, and TMAO, can activate Toll-like receptors, the NLRP3 inflammasome, and nucleic acid–sensing pathways, thereby promoting inflammatory cytokine production, endothelial dysfunction, and foam cell formation. In contrast, beneficial microbial metabolites such as short-chain fatty acids, bile acids, and tryptophan-derived metabolites exert immunomodulatory and vasculoprotective effects through signaling pathways involving FFAR2/3, the AhR, the FXR, and TGR5. Conversely, the innate immune system shapes microbial composition and function through barrier defense, phagocytic clearance, and antimicrobial factor production, establishing a dynamic and reciprocal microbiota–immune interaction network. This review systematically summarizes alterations in microbial ecology and innate immune homeostasis associated with atherosclerosis, elucidates the key molecular mechanisms underlying microbiota–innate immune crosstalk, and examines its dynamic involvement across four critical stages of disease evolution: endothelial dysfunction, foam cell formation, plaque progression, and plaque destabilization and rupture. In addition, emerging therapeutic approaches, including microbiota remodeling, modulation of microbial metabolic pathways, and precision microbiome-based interventions, are comprehensively discussed. The microbiota–innate immune axis provides a novel conceptual framework for understanding atherosclerosis pathogenesis and represents a promising target for future disease prevention, risk stratification, and precision therapeutics.

## 1 Introduction

Atherosclerosis (AS) is the primary pathological basis underlying major cardiovascular diseases, including coronary artery disease, ischemic stroke, and peripheral arterial disease, and remains a leading cause of morbidity and mortality worldwide (1, 2). The development of AS is a complex and multifactorial process characterized by endothelial dysfunction, lipid accumulation, foam cell formation, inflammatory cell infiltration, and the progressive formation and destabilization of atherosclerotic plaques. These pathological changes can ultimately precipitate severe cardiovascular events such as myocardial infarction and stroke (3–5). Traditionally, AS has been regarded as a disease driven predominantly by classical risk factors, including dyslipidemia, hypertension, smoking, diabetes mellitus, and genetic susceptibility (6, 7). However, these factors alone cannot fully account for the substantial interindividual variability and clinical heterogeneity observed in disease onset, progression, and outcomes. Increasing evidence suggests that atherosclerosis is not merely a consequence of lipid deposition and vascular injury but also a chronic inflammatory disorder accompanied by immune dysregulation and alterations in microbial ecology (8).

The microbiota plays a pivotal role in maintaining host metabolic homeostasis, barrier integrity, and immune balance (9). Microbial-derived molecules, particularly lipopolysaccharide (LPS), can activate signaling pathways such as Toll-like receptor 4 (TLR4)/nuclear factor-κB (NF-κB) and the NLR family pyrin domain-containing 3 (NLRP3) inflammasome, thereby promoting the production of pro-inflammatory cytokines, including interleukin-1β (IL-1β), tumor necrosis factor-α (TNF-α), and IL-6, and amplifying vascular inflammatory responses (10, 11). Conversely, beneficial microbial metabolites such as short-chain fatty acids (SCFAs) can modulate macrophage polarization and regulate immune homeostasis, thereby influencing atherosclerosis-associated immune remodeling (12). Importantly, the relationship between the microbiota and innate immunity is bidirectional. Innate immune mechanisms—including epithelial barrier defense, antimicrobial peptide secretion, cytokine production, and phagocyte-mediated microbial clearance—continuously shape microbial composition and function (13, 14). Under atherosclerotic conditions, however, this regulatory capacity becomes dysregulated, potentially leading to further microbial imbalance and establishing a self-reinforcing cycle of dysbiosis and aberrant innate immune activation.

Notably, recent high-impact studies have provided emerging causal evidence suggesting that interactions between the gut microbiota and the immune system are not only closely associated with the initiation and progression of atherosclerosis but may also exert a direct driving role in disease development (15). However, most current research has primarily focused on the mechanistic effects of either microbial factors or innate immune components in isolation. A comprehensive understanding of the interactive networks between these two systems, as well as their dynamic regulation across different stages of atherosclerosis, remains insufficient. Accordingly, this review systematically summarizes microbiota-associated ecological alterations and innate immune dysregulation in atherosclerosis, with a particular focus on the key molecular mechanisms underlying microbiota–innate immune crosstalk. In addition, we integrate disease-stage-specific dynamics to delineate their temporal evolution and summarize recent advances in therapeutic interventions targeting this axis. From a mechanistic integration perspective, this work aims to provide new insights into the pathophysiology of atherosclerosis and to identify potential therapeutic targets for future intervention strategies.

## 2 Microbial ecological alterations and disruption of innate immune homeostasis in atherosclerosis

In recent years, advances in metagenomics, metabolomics, and single-cell omics technologies have enabled a more systematic characterization of microbiota-associated alterations in atherosclerosis (16–18). Compared with healthy individuals, patients with atherosclerosis consistently exhibit substantial remodeling of both microbial composition and functional capacity; however, the specific patterns of these changes are highly heterogeneous (19). Differences in dominant microbial taxa and metabolic profiles across studies may be attributed to variations in dietary patterns, age, host metabolic status, disease stage, and medication exposure. Therefore, relying solely on taxonomic abundance is insufficient to fully explain the role of the microbiota in atherosclerosis. Current research increasingly emphasizes ecological function, metabolic network interactions, and host immune adaptation as more comprehensive frameworks for understanding microbial dysbiosis in this disease context.

At the ecological level, microbiota-associated alterations in atherosclerosis are closely linked to remodeling of the host metabolic microenvironment. High-fat diet, obesity, insulin resistance, and chronic inflammatory states can reshape microbial community structure and metabolic activity, thereby promoting the sustained enrichment of specific microbial populations and their bioactive metabolites (20–22). In parallel, alterations in bile acid metabolism, lipid metabolism, and oxidative stress further perturb microbial ecological homeostasis (23, 24). Such niche remodeling not only affects microbial stability but also increases the likelihood of interactions between microbial-derived signals and host tissues or immune cells, thereby providing persistent stimuli that drive chronic inflammation and immune remodeling.

At the barrier level, microbial dysbiosis can compromise both intestinal and vascular barrier integrity. Reduced expression of tight junction proteins, disruption of the mucus layer, and impaired epithelial repair capacity collectively contribute to increased intestinal permeability, facilitating the translocation of lipopolysaccharide and other microbial-associated molecular patterns into the systemic circulation (25–27). Rather than manifesting as acute inflammation, this barrier dysfunction is more commonly associated with sustained low-grade immune activation and chronic inflammatory responses (28). This state promotes aberrant interactions among endothelial cells, immune cells, and vascular wall components, thereby creating a permissive environment for atherosclerotic lesion formation (29).

At the immune level, innate immune homeostasis is also markedly altered. Under physiological conditions, barrier defenses, antimicrobial peptide secretion, phagocytic clearance, and immune tolerance mechanisms collectively maintain a dynamic equilibrium between the host and the microbiota (13, 30). In atherosclerosis-associated states, however, this balance becomes progressively disrupted. Innate immune populations, particularly macrophages and neutrophils, undergo functional reprogramming characterized by persistent cytokine release, hyperactivation of pattern-recognition receptors, impaired phagocytic capacity, and enhanced pro-inflammatory responses (31, 32).

Therefore, microbial dysbiosis provides sustained stimuli that drive chronic inflammation and immune remodeling in atherosclerosis, whereas metabolic disturbances, vascular inflammation, and immune stress in turn selectively shape specific microbial communities. Recent studies have further suggested that microbiota-driven metabolic reprogramming and immune cell functional remodeling are tightly coupled and may occur synchronously across multiple cardiovascular diseases, highlighting the integrated and systems-level nature of this regulatory network (33). Importantly, most existing evidence remains largely associative, and the temporal dynamics and causal mechanisms linking microbial ecological alterations to innate immune remodeling remain to be fully elucidated.

## 3 Molecular mechanisms and signaling regulation underlying microbiota–innate immune crosstalk

**Figure 1:**
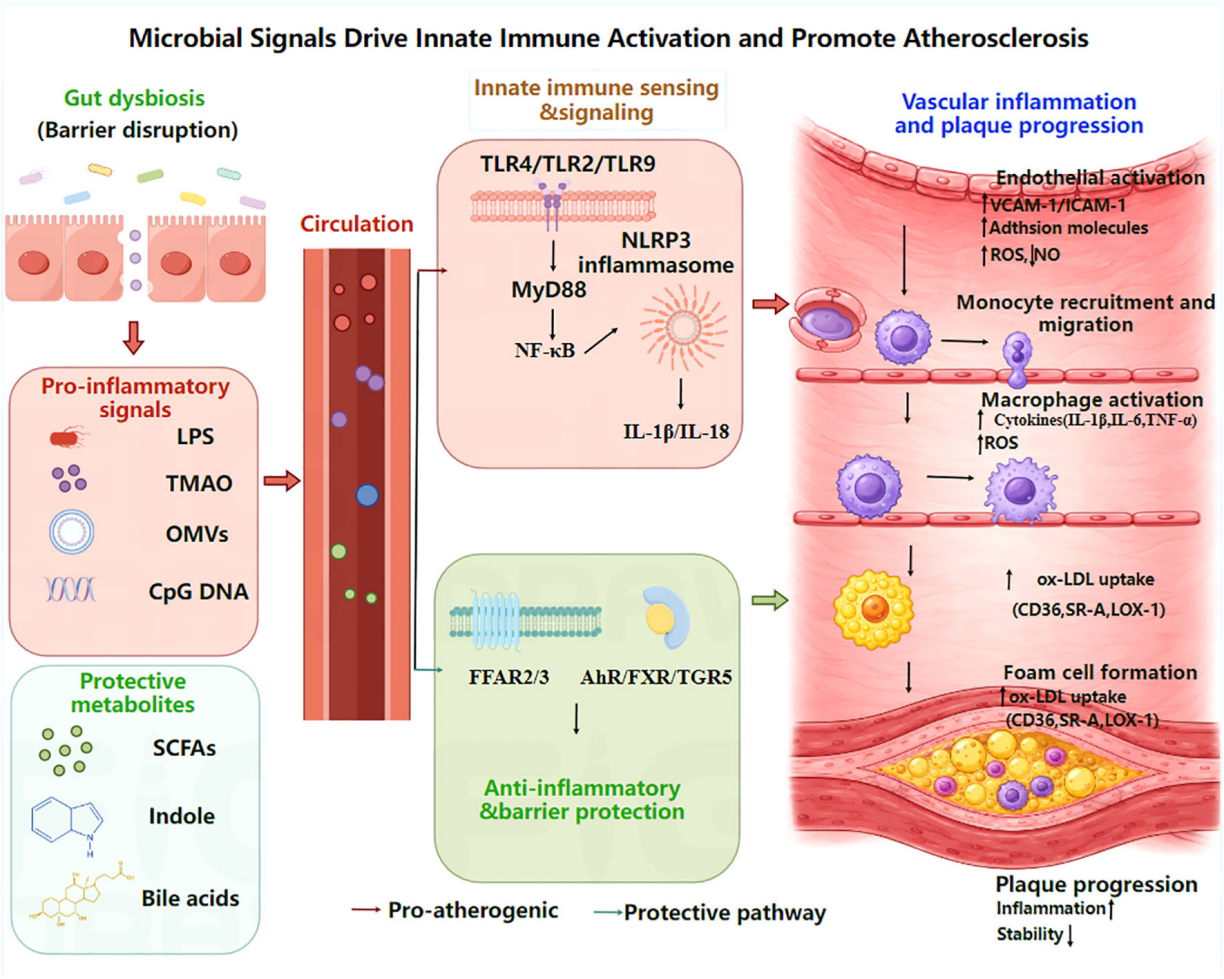
Mechanisms of microbiota–innate immunity crosstalk in atherosclerosis. Mechanisms of microbiota–innate immunity crosstalk in atherosclerosis. Gut microbial dysbiosis and barrier dysfunction facilitate the translocation of microbial-derived signals, including LPS, TMAO, OMVs, and CpG DNA, into the circulation. These signals activate innate immune sensing pathways, primarily through Toll-like receptors, MyD88-dependent signaling, NF-κB, and the NLRP3 inflammasome, resulting in increased production of pro-inflammatory cytokines such as IL-1β, IL-6, TNF-α, and IL-18. Subsequently, endothelial activation, monocyte recruitment, macrophage activation, and foam cell formation contribute to plaque progression. In contrast, protective microbial metabolites, including SCFAs, indole derivatives, and bile acids, exert anti-inflammatory and barrier-protective effects through FFAR2/3, AhR, FXR, and TGR5, thereby counteracting atherogenic inflammation.

Microorganisms regulate innate immune function through multiple classes of signals, including structural components, metabolic products, secreted factors, and extracellular nucleic acids (**Figure 1**). These signals exhibit a hierarchical organization in terms of their immunological functions. Microbial structural components primarily mediate immune recognition through pattern-recognition receptors and initiate downstream inflammatory cascades. In contrast, microbial metabolites are more closely involved in immune cell differentiation, metabolic reprogramming, and the regulation of inflammatory microenvironments (34). In addition, microbial secretory factors, outer membrane vesicles, and nucleic acid–associated molecules contribute to signal amplification, intercellular communication, and long-distance immune modulation. Conversely, the innate immune system can reciprocally shape microbial composition, spatial distribution, and metabolic activity, thereby forming a continuously dynamic and bidirectional interaction network.

### 3.1 Molecular mechanisms by which microorganisms regulate innate immunity

#### 3.1.1 Microbial structural components

Microbial structural components represent fundamental ligands recognized by the innate immune system and include lipopolysaccharide (LPS), lipoteichoic acid, peptidoglycan, and flagellin (35). These molecules play critical roles in regulating immune homeostasis and vascular inflammatory responses (36). Emerging evidence suggests that innate immune activation triggered by microbial structural components constitutes a key mechanistic link between microbial dysbiosis and atherosclerosis development (37).

Among these components, LPS derived from Gram-negative bacteria is the most extensively studied. Following impairment of intestinal barrier integrity, circulating LPS can access the vascular compartment and act on endothelial cells, monocytes, and macrophages (38). Through activation of the TLR4/MyD88/NF-κB signaling axis, LPS induces the expression of pro-inflammatory mediators, including interleukin-1β, interleukin-6, tumor necrosis factor-α, and monocyte chemoattractant proteins (39–41). In addition, LPS upregulates endothelial adhesion molecules, thereby promoting monocyte recruitment to the vascular wall and facilitating foam cell formation and plaque progression (42). Sustained LPS exposure can further trigger activation of the NLR family pyrin domain-containing 3 inflammasome, leading to maturation and release of interleukin-1β and interleukin-18 and amplification of local inflammatory responses (43).

Beyond LPS, lipoteichoic acid and peptidoglycan derived from Gram-positive bacteria signal through pattern-recognition receptors such as Toll-like receptor 2, nucleotide-binding oligomerization domain-containing protein 1, and nucleotide-binding oligomerization domain-containing protein 2, thereby modulating the functions of monocytes, macrophages, and dendritic cells and enhancing vascular inflammation (44, 45). Flagellin is recognized by Toll-like receptor 5, activating downstream NF-κB signaling and influencing endothelial activation as well as immune cell recruitment (46). Recent studies have also highlighted that structural components derived from oral pathogens contribute to atherosclerosis development by inducing inflammatory responses, promoting oxidative stress, and disrupting lipid metabolism (47).

In contrast, structural components derived from commensal microorganisms may exert immunoregulatory effects. For instance, bacterial surface polysaccharides can promote tolerance-associated signaling pathways and restrain excessive inflammatory activation and tissue injury (48). However, under conditions of atherosclerosis-associated dysbiosis, this homeostatic regulation is progressively disrupted, and microbial signals that normally maintain immune equilibrium may instead become key drivers of chronic inflammation, foam cell formation, and plaque progression.

#### 3.1.2 Microbial metabolites

Unlike microbial structural components, which primarily activate pattern-recognition receptor signaling, microbial metabolites exert more direct effects on host immunometabolism, inflammatory responses, and vascular pathology. Many of these metabolites can cross the intestinal barrier and enter the circulation, where they influence immune cell function, intracellular signaling pathways, and metabolic states, thereby contributing to innate immune remodeling during atherosclerosis. Major microbial metabolites and their immunological effects in atherosclerosis are summarized in **Table 1**.

**Table 1:** Immunomodulatory effects of microbial-derived metabolites on innate immune responses in atherosclerosis.

Metabolite	Target cells	Main pathways	Effects in atherosclerosis (AS)	References
SCFAs				
Acetate	Macrophages, endothelial cells	FFAR2/GPR43, AMPK	Suppresses inflammatory cytokine release, improves endothelial dysfunction, and attenuates vascular inflammation in AS lesions	(49, 50)
Propionate	Dendritic cells, Treg cells	FFAR2/3, HDAC inhibition	Enhances immune tolerance and maintains Treg homeostasis, thereby suppressing chronic vascular inflammation	(51, 52)
Butyrate	Macrophages, endothelial cells	HDAC inhibition, FFAR2/3	Inhibits foam cell formation, reduces inflammatory burden, and promotes plaque stability	(53, 54)
Valerate	Macrophages, T cells	HDAC inhibition	Regulates immune cell differentiation and inflammatory balance, contributing to immunometabolic remodeling in AS	(55, 56)
Bile acid metabolites				
Primary bile acids	Hepatocytes, immune cells	FXR	Regulate cholesterol metabolism and systemic inflammation, indirectly influencing AS progression	(57, 58)
Deoxycholic acid (DCA)	Macrophages	FXR/TGR5, ROS	Promotes oxidative stress and inflammation, accelerating plaque progression	(59, 60)
Lithocholic acid (LCA)	Treg cells, dendritic cells	VDR, TGR5	Maintains immune homeostasis and suppresses excessive inflammation in vascular microenvironment	(61, 62)
Ursodeoxycholic acid (UDCA)	Endothelial cells, macrophages	FXR/TGR5, antioxidant pathways	Attenuates oxidative stress and endothelial injury, slowing AS progression	(63, 64)
Tryptophan metabolites				
Indole and derivatives	Endothelial cells, macrophages	AhR	Maintain gut–vascular barrier integrity and suppress inflammatory activation in AS	(65, 66)
Indole-3-propionic acid (IPA)	Macrophages, endothelial cells	AhR, PXR	Exerts antioxidant effects and reduces vascular inflammation and endothelial injury	(67, 68)
Indole-3-acetic acid (IAA)	Myeloid cells	AhR	Modulates immunometabolic balance and inflammatory response intensity	(69, 70)
Kynurenine	Monocytes, T cells	IDO1–AhR axis	Promotes immune tolerance imbalance and sustains chronic inflammation in AS	(71, 72)
Choline-related metabolites				
Trimethylamine (TMA)	Macrophages	FMO3 metabolic pathway	Participates in host metabolic reprogramming and promotes vascular inflammation	(73, 74)
Trimethylamine N-oxide (TMAO)	Macrophages, endothelial cells	TLR4/NF-κB, NLRP3 inflammasome	Promotes foam cell formation, endothelial dysfunction, and plaque progression	(75)
Other metabolites				
Succinate	Macrophages	HIF-1α stabilization, metabolic reprogramming	Accumulates in AS lesions and drives pro-inflammatory macrophage polarization with IL-1β production	(76, 77)
Lactate	Macrophages, endothelial cells	HIF-1α-dependent metabolic reprogramming	Promotes immunometabolic adaptation and endothelial inflammation under hypoxic conditions	(78, 79)
ATP	Macrophages	P2X7/NLRP3 inflammasome	Acts as a DAMP to activate inflammasomes and amplify IL-1β/IL-18-mediated inflammation	(80, 81)
Adenosine	Macrophages, neutrophils	A2A/A2B receptors	Suppresses excessive myeloid activation and maintains immune homeostasis	(82, 83)
Hydrogen sulfide (H_2_S)	Endothelial cells, vascular smooth muscle cells	Nrf2 antioxidant pathway	Reduces oxidative stress, improves endothelial function, and regulates vascular tone	(84, 85)

Short-chain fatty acids represent the most extensively studied class of microbial metabolites. Acetate, propionate, butyrate, and valerate regulate the functions of macrophages, dendritic cells, and endothelial cells through free fatty acid receptors 2 and 3, G protein-coupled receptor 43 signaling, and histone deacetylase inhibition. Among them, butyrate has received particular attention because it suppresses inflammatory cytokine production, reduces foam cell formation, and promotes plaque stability, thereby contributing to the maintenance of vascular immune homeostasis.

Bile acid metabolites serve as important signaling intermediates linking host metabolism, microbial ecology, and immune regulation. Secondary bile acids, including deoxycholic acid, lithocholic acid, and ursodeoxycholic acid, regulate macrophage polarization, inflammatory responses, and cholesterol metabolism through receptors such as the farnesoid X receptor, Takeda G protein-coupled receptor 5, and vitamin D receptor. While deoxycholic acid has been associated with enhanced inflammatory activity and accelerated plaque progression, other bile acid species exhibit anti-inflammatory and vasculoprotective properties, highlighting the context-dependent nature of bile acid signaling.

Tryptophan-derived metabolites primarily influence immune regulation through aryl hydrocarbon receptor–dependent pathways. Indole and its derivatives contribute to barrier maintenance and suppression of inflammatory responses, whereas excessive activation of the kynurenine pathway may promote chronic inflammation and immune dysregulation. Among microbial metabolites associated with atherosclerosis, trimethylamine N-oxide has attracted particular interest because of its ability to activate TLR4/NF-κB signaling and the NLR family pyrin domain-containing 3 inflammasome, thereby promoting macrophage activation, foam cell formation, and plaque progression.

In addition to the major microbial metabolites described above, succinate, lactate, adenosine triphosphate (ATP), and adenosine also play important roles in the regulation of atherosclerosis-associated immune responses. Within the atherosclerotic microenvironment, succinate and ATP generally function as pro-inflammatory metabolic signals. They enhance the activation state of macrophages and endothelial cells, promote NLR family pyrin domain-containing 3 inflammasome activation, and facilitate the release of pro-inflammatory cytokines, thereby exacerbating local vascular inflammation. In contrast, adenosine exerts predominantly immunosuppressive effects through activation of A2A and A2B receptors, thereby limiting excessive immune activation and contributing to the maintenance of vascular immune homeostasis.

Collectively, microbial-derived metabolites regulate atherosclerosis progression through multiple interconnected mechanisms, including immunometabolic reprogramming, modulation of inflammatory signaling pathways, and regulation of vascular cellular function. A more comprehensive understanding of the interactions between these metabolic signals and the innate immune system may facilitate the identification of novel therapeutic targets and support the development of precision microbiome-based interventions for atherosclerosis.

#### 3.1.3 Microbial secretory factors and nucleic acid–associated molecules

In addition to structural components and metabolites, microorganisms can modulate atherosclerosis-related immune responses through a variety of bioactive secreted factors. These include outer membrane vesicles (OMVs), secreted proteins, extracellular DNA and RNA, and other nucleic acid–associated signals (86, 87). Compared with conventional microbial components, these molecules possess enhanced capacity for long-distance communication, enabling them to traverse local barriers and enter the circulation, where they exert systemic effects on vascular and immune microenvironments (86).

OMVs represent a key mechanism of intercellular communication in host–microbe interactions. These nanoscale vesicles transport diverse bioactive cargo, including proteins, lipids, and nucleic acids, thereby facilitating the direct delivery of microbial signals to host cells. Following uptake by macrophages, OMVs activate inflammatory signaling cascades such as the TLR4/NF-κB pathway, leading to enhanced cytokine production and immune cell activation (88). In addition, OMVs amplify oxidative stress responses, impair endothelial stability, and promote the establishment of a pro-inflammatory vascular microenvironment (89).

Microbial nucleic acid–associated signals constitute another critical interface linking dysbiosis to innate immune activation. CpG motifs within bacterial DNA are recognized by Toll-like receptor 9, resulting in downstream inflammatory signaling activation (90). Moreover, extracellular DNA and RNA released from microorganisms or damaged host cells can engage cytosolic nucleic acid–sensing pathways, including the cyclic GMP–AMP synthase–stimulator of interferon genes axis, thereby amplifying innate immune responses and sustaining inflammatory activation (91).

### 3.2 Regulation of microbial ecology by innate immunity

The innate immune system not only responds to microbial-derived signals but also plays an active role in maintaining microbial ecological homeostasis. Under physiological conditions, host defense mechanisms—including barrier integrity, phagocytic clearance, and inflammatory signaling regulation—collectively govern microbial composition, spatial distribution, and metabolic activity, thereby preserving host–microbiota equilibrium (92).

Barrier systems represent a fundamental component of innate immune control over microbial ecology. The intestinal epithelium maintains microbial segregation through tight junction proteins, the mucus layer, and antimicrobial molecules (93). When barrier integrity is preserved, most microorganisms remain confined to the intestinal lumen, thereby limiting direct interactions with host tissues and immune cells (94). However, in the context of atherosclerosis-associated chronic inflammation and metabolic dysfunction, barrier integrity is progressively compromised, allowing microbial-derived signals to translocate into the circulation and further exacerbate inflammatory and immune disturbances (95).

Phagocytic cells also play essential roles in maintaining microbial homeostasis. Macrophages, neutrophils, and dendritic cells restrict excessive microbial expansion through phagocytosis, reactive oxygen species production, and cytokine secretion (96). Simultaneously, pattern-recognition receptors continuously sense microbial signals and dynamically adjust immune responses according to microenvironmental cues, thereby influencing microbial community structure and metabolic output (97, 98). When innate immune regulation becomes dysregulated, this homeostatic control is weakened, contributing to further microbial imbalance.

Antimicrobial peptides provide an additional mechanistic link between innate immunity and microbial ecology. Epithelial and immune cells produce defensins, lysozyme, and related antimicrobial factors that selectively shape microbial populations (99, 100). During chronic inflammatory states, alterations in antimicrobial peptide expression profiles can disrupt microbial stability and metabolic function (101). In parallel, persistent inflammatory signaling reshapes the local nutrient milieu, redox balance, and metabolic environment, further influencing microbial ecological composition (102).

Collectively, microorganisms and innate immunity form a tightly interconnected regulatory network mediated by barrier function, phagocytic activity, antimicrobial factors, and inflammatory signaling pathways. Within the context of atherosclerosis, disruption of innate immune homeostasis is not only a consequence of microbial dysbiosis but may also actively drive microbial ecological remodeling. Through reciprocal reinforcement, these processes establish a self-amplifying loop that sustains chronic inflammation and promotes the progression of atherosclerosis.

## 4 Dynamic evolution of microbiota–innate immune crosstalk during atherosclerosis progression

**Figure 2:**
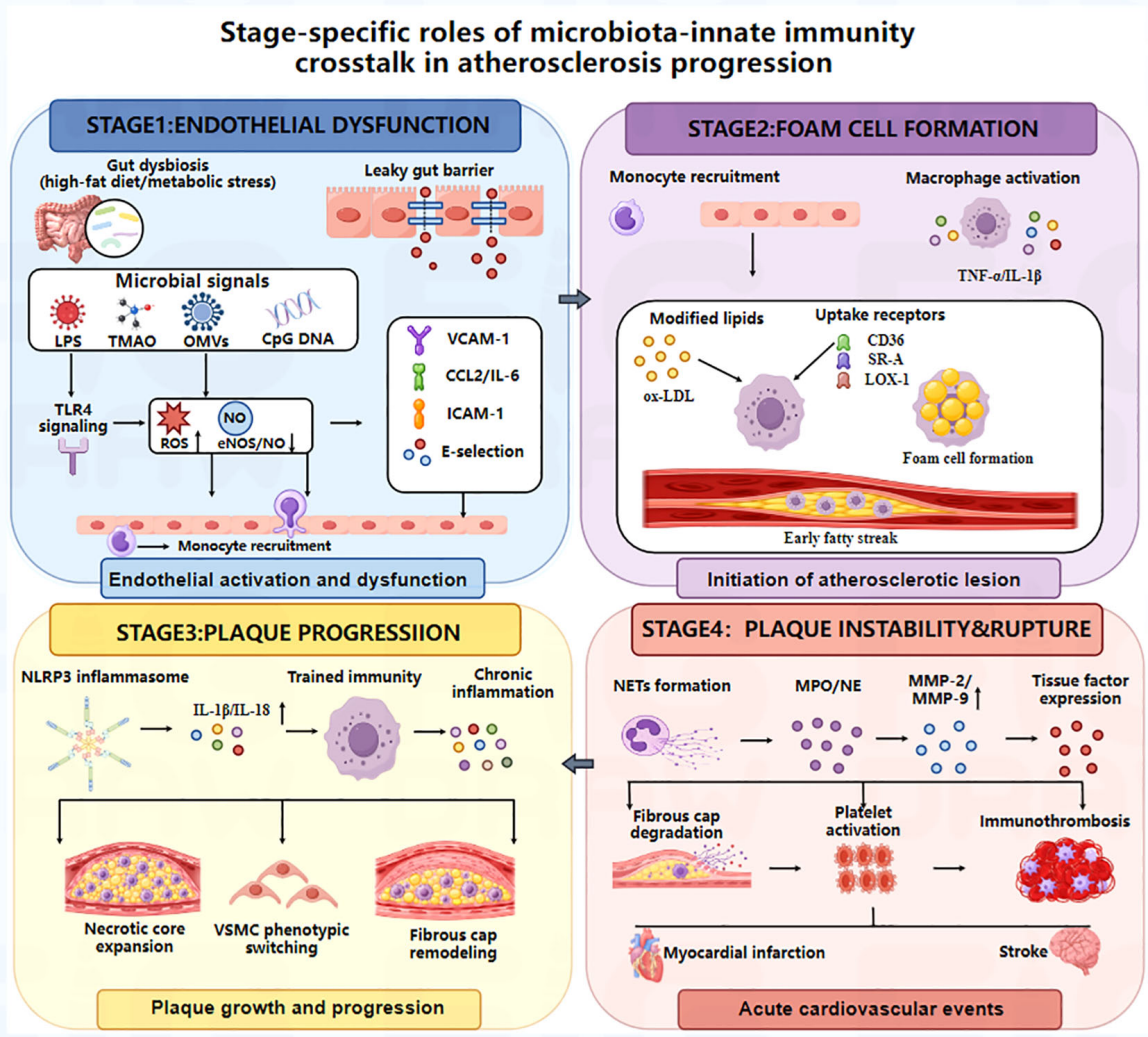
Dynamic evolution of microbiota–innate immunity crosstalk during atherosclerosis progression. The contribution of microbiota–innate immunity interactions evolves throughout the course of atherosclerosis. During the endothelial dysfunction stage, microbial dysbiosis and gut barrier disruption promote the systemic dissemination of microbial signals, leading to endothelial activation and monocyte recruitment through increased expression of VCAM-1, ICAM-1, E-selectin, and pro-inflammatory mediators. During foam cell formation, macrophages exhibit enhanced uptake of ox-LDL via scavenger receptors including CD36, SR-A, and LOX-1, resulting in lipid accumulation and early lesion development. In the plaque progression stage, chronic activation of the NLRP3 inflammasome, sustained production of IL-1β and IL-18, and trained immunity promote necrotic core expansion, VSMC phenotypic switching, and fibrous cap remodeling. During plaque instability and rupture, NETs, matrix metalloproteinases, and tissue factor-mediated immunothrombosis accelerate plaque destabilization and contribute to acute cardiovascular events such as myocardial infarction and ischemic stroke.

The interaction between microorganisms and innate immunity is not a static phenomenon but rather a dynamic process that spans the entire course of atherosclerosis, from disease initiation to the occurrence of adverse cardiovascular events. At different stages of disease progression, distinct microbial signals and innate immune responses become dominant, collectively influencing endothelial injury, lipid accumulation, foam cell formation, plaque growth, and plaque stability. As atherosclerosis advances, microbial dysbiosis and innate immune dysfunction progressively evolve from localized defensive responses into self-sustaining inflammatory drivers that ultimately promote plaque rupture and thrombus formation. Therefore, understanding microbiota–innate immune crosstalk from a disease-evolution perspective may provide important insights into the mechanisms underlying persistent disease progression (**Figure 2**).

### 4.1 Endothelial dysfunction stage

Endothelial dysfunction is widely regarded as the initiating event in atherosclerosis and represents not merely vascular injury but an early disruption of vascular immune homeostasis (103). Under physiological conditions, the vascular endothelium serves not only as a physical barrier but also as an active regulator of local immune tolerance. Through the production of nitric oxide and other protective mediators, endothelial cells maintain anti-inflammatory and anti-adhesive properties that limit prolonged interactions between circulating immune cells and the vessel wall (104, 105). However, when microbial dysbiosis and metabolic disturbances develop, this immune equilibrium gradually deteriorates, transforming the endothelium from a tolerogenic barrier into an active immunological sensing interface.

Accumulating evidence suggests that microbial-derived signals play a pivotal role in driving this transition. Following impairment of intestinal barrier integrity, lipopolysaccharide, peptidoglycan, and bacterial extracellular vesicles gain access to the circulation, where they are continuously detected by endothelial cells and myeloid immune populations (25, 26). Among these factors, lipopolysaccharide promotes vascular inflammation through Toll-like receptor 4–dependent signaling, inducing the expression of vascular cell adhesion molecule 1, intercellular adhesion molecule 1, and E-selectin. Consequently, CCR2-positive monocytes that normally exhibit limited vascular adhesion progressively accumulate along the endothelial surface (106, 107). Simultaneously, trimethylamine N-oxide enhances reactive oxygen species generation and suppresses endothelial nitric oxide synthase activity, thereby reducing nitric oxide bioavailability and further compromising endothelial protective functions (108, 109). Under these conditions, endothelial cells become not only recipients of inflammatory stimuli but also active producers of chemokines and cytokines, including CCL2, CXCL1, and interleukin-6, which further recruit innate immune cells to sites of vascular injury.

Importantly, the most critical event during this stage is not the transient elevation of inflammatory mediators but the acquisition of a sustained capacity for immune cell recruitment by the vascular wall. Monocytes that infiltrate the intima become progressively activated within the local inflammatory milieu and release effector molecules such as interleukin-1β, tumor necrosis factor-α, and reactive oxygen species (110). These mediators exacerbate endothelial injury and oxidative stress (111) while simultaneously impairing intestinal barrier integrity and altering microbial community composition. Such changes favor the expansion of trimethylamine-producing and other pro-inflammatory microbial populations, thereby increasing the burden of circulating microbial-derived signals (112). As a result, innate immune activation induced by microbial dysbiosis further amplifies microbial abnormalities, establishing a self-reinforcing microbiota–immune positive feedback loop.

Therefore, the endothelial dysfunction stage should not be viewed merely as a passive starting point of atherosclerosis. Rather, it represents a critical pathological checkpoint at which microbial dysbiosis and innate immune dysregulation first become mechanistically coupled. During this phase, the vascular wall progressively shifts from an immune-tolerant tissue toward a chronic inflammatory platform capable of continuously recruiting and activating immune cells, thereby laying the foundation for subsequent lipid accumulation, macrophage infiltration, and foam cell formation.

### 4.2 Foam cell formation stage

Foam cell formation represents a pivotal step in the transition from early vascular inflammation to the development of lipid-rich atherosclerotic plaques and marks a critical stage at which microbiota–innate immune crosstalk shifts from inflammatory initiation to lesion amplification. Following endothelial dysfunction, large amounts of low-density lipoprotein infiltrate the intima and undergo oxidative modification, generating oxidized low-density lipoprotein (113, 114). Concurrently, recruited monocytes progressively differentiate into macrophages and become exposed to a local microenvironment enriched in oxidized low-density lipoprotein, cholesterol crystals, and inflammatory mediators (115). At this stage, the primary impact of microbiota–innate immune interactions gradually shifts from endothelial activation toward the regulation of macrophage lipid metabolism and inflammatory reprogramming.

Microbial-derived signals and the inflammatory milieu they generate promote the expression of scavenger receptors, including cluster of differentiation 36, scavenger receptor A, and lectin-like oxidized low-density lipoprotein receptor 1, thereby markedly enhancing macrophage uptake of oxidized lipoproteins (116). At the same time, cholesterol efflux pathways mediated by ATP-binding cassette transporter A1, ATP-binding cassette transporter G1, and apolipoprotein A-I become impaired, resulting in persistent intracellular accumulation of cholesterol esters (117, 118). As lipid burden progressively increases, macrophages undergo phenotypic transformation into foam cells, which subsequently become one of the dominant inflammatory effector populations within atherosclerotic lesions (119).

Importantly, foam cell formation is not merely a process of lipid deposition but is accompanied by profound functional reprogramming of macrophages. Excessive lipid accumulation induces mitochondrial dysfunction, enhances reactive oxygen species production, and alters cellular metabolic programs, driving macrophages from a homeostatic state toward a pro-inflammatory phenotype (120). Foam cells continuously release interleukin-1β, tumor necrosis factor-α, interleukin-6, CCL2, and other chemotactic mediators, thereby promoting further monocyte recruitment and sustaining local inflammatory responses. This process establishes a pathological cycle in which lipid accumulation and inflammatory amplification mutually reinforce one another (121).

As lesions progress, foam cell apoptosis becomes increasingly prevalent. Simultaneously, defective efferocytosis compromises the clearance of dying cells, resulting in secondary necrosis and the release of cholesterol crystals, oxidized lipids, and damage-associated molecular patterns (122). These danger signals further activate surrounding innate immune cells, perpetuating inflammation and accelerating lesion expansion. Consequently, foam cell formation serves as a critical bridge linking early endothelial dysfunction to the development of advanced atherosclerotic plaques.

### 4.3 Plaque progression stage

With the continued accumulation of foam cells and progressive intensification of local inflammation, atherosclerotic lesions evolve from simple lipid deposits into structurally complex and biologically active plaques. This stage is characterized by necrotic core formation, persistent chronic inflammation, phenotypic switching of vascular smooth muscle cells, and extensive extracellular matrix remodeling (123). Compared with earlier stages of disease, microbiota–innate immune crosstalk during plaque progression extends beyond disturbances in lipid metabolism and is increasingly dominated by sustained inflammation and dysregulated tissue remodeling.

The continuous release of cholesterol crystals from expanding lesions further activates macrophages and dendritic cells, thereby amplifying local inflammatory responses (5). Simultaneously, cholesterol crystals and cellular debris induce persistent activation of the NLR family pyrin domain-containing 3 inflammasome, promoting the maturation and secretion of interleukin-1β and interleukin-18 and driving the transition from localized inflammation to chronic innate immune activation (124).

Notably, trained immunity has emerged as an important mechanism linking microbial exposure to sustained atherosclerotic progression (125). Following prolonged exposure to lipopolysaccharide, oxidized lipids, and other microbe-associated metabolic signals, monocytes and macrophages undergo epigenetic reprogramming and metabolic remodeling, resulting in heightened responsiveness to subsequent stimuli (27). This phenomenon, often referred to as inflammatory memory, enables innate immune cells to maintain enhanced production of inflammatory mediators such as interleukin-1β, tumor necrosis factor-α, and interleukin-6 even after the initial stimulus has diminished (126). Consequently, microbial-derived signals no longer act merely as transient inflammatory triggers but instead promote long-term disease progression through persistent reprogramming of innate immune function.

In addition to immune cells, vascular smooth muscle cells are major contributors to plaque development. Under the combined influence of microbial-associated signals and inflammatory mediators, these cells gradually transition from a contractile phenotype to a synthetic phenotype and migrate into the intima, where they participate in fibrous cap formation and extracellular matrix deposition (127). However, persistent inflammation simultaneously promotes matrix degradation and disrupts smooth muscle cell function, leading to increasing structural complexity and vulnerability of the plaque (128). Moreover, local hypoxia, metabolic dysregulation, and ongoing inflammatory cell infiltration further accelerate necrotic core expansion, thereby creating conditions that favor subsequent plaque destabilization (129).

The defining pathological feature of the plaque progression stage is the establishment of a long-term positive feedback network involving microbial-derived signals, sustained innate immune activation, and vascular wall remodeling. Through the promotion of chronic inflammation, necrotic core enlargement, and smooth muscle cell phenotypic switching, microbiota–innate immune crosstalk drives the evolution of atherosclerosis from early lesions to high-risk mature plaques.

### 4.4 Plaque destabilization and rupture stage

Plaque destabilization and rupture constitute the pathological basis of acute cardiovascular and cerebrovascular events, including myocardial infarction, ischemic stroke, and sudden vascular death (1). Stable plaques are generally characterized by a thick fibrous cap and relatively limited inflammatory infiltration, whereas vulnerable plaques exhibit an enlarged necrotic core, thinning of the fibrous cap, and persistent accumulation of inflammatory cells (123). Following prolonged microbiota–innate immune crosstalk, the pathological landscape gradually shifts from chronic inflammation toward progressive tissue destruction and a prothrombotic state.

Macrophages remain central effector cells during this stage. Persistent exposure to microbial-derived signals and inflammatory stimuli sustains macrophage activation and promotes the expression of matrix metalloproteinases, particularly matrix metalloproteinase-2 and matrix metalloproteinase-9 (130, 131). These proteolytic enzymes degrade collagen fibers and extracellular matrix components, thereby weakening the structural integrity of the fibrous cap (125). Concurrently, the inflammatory milieu induces vascular smooth muscle cell apoptosis and suppresses collagen synthesis, reducing the capacity for fibrous cap repair and stabilization (123). As matrix degradation progressively exceeds tissue repair, plaques acquire increasingly vulnerable characteristics.

Neutrophils also play a crucial role in plaque destabilization. Vulnerable plaques exhibit markedly increased neutrophil infiltration, and the formation of neutrophil extracellular traps contributes not only to local inflammatory amplification but also to endothelial injury and macrophage activation (132). Neutrophil elastase and myeloperoxidase released from these extracellular structures further degrade fibrous cap components and enhance oxidative stress, thereby accelerating plaque vulnerability (133, 134). In addition to their direct tissue-destructive effects, neutrophil extracellular traps serve as important mediators linking inflammation and thrombosis during advanced disease.

Following plaque rupture or superficial erosion, cholesterol crystals, tissue factor, and other procoagulant components contained within the necrotic core become exposed to circulating blood, triggering rapid platelet activation and coagulation cascade initiation (133). Simultaneously, neutrophil extracellular traps provide a scaffold for platelet adhesion and fibrin deposition, whereas activated platelets release high-mobility group box 1, CXC motif chemokine ligand 4, and P-selectin, which further promote neutrophil extracellular trap formation. This reciprocal interaction establishes a characteristic process known as immunothrombosis (135). The amplification network formed among macrophages, neutrophils, and platelets not only accelerates local thrombus formation but is also widely recognized as a central pathological mechanism underlying acute myocardial infarction and ischemic stroke.

Taken together, microbiota–innate immune crosstalk exhibits a continuous and progressively evolving pattern throughout atherosclerosis development. During the early phase, it is primarily characterized by disruption of endothelial immune homeostasis and recruitment of circulating monocytes. Subsequently, it promotes macrophage lipid dysregulation, foam cell formation, and inflammatory amplification. As disease advances, the interaction network contributes to necrotic core expansion, maintenance of trained immunity, and vascular wall remodeling. Ultimately, it drives fibrous cap degradation, neutrophil extracellular trap formation, and immunothrombosis. These findings collectively highlight the microbiota–innate immune axis as a fundamental regulatory network that operates throughout the entire disease course and orchestrates transitions between pathological stages.

## 5 Therapeutic strategies targeting the microbiota–innate immune axis

**Figure 3:**
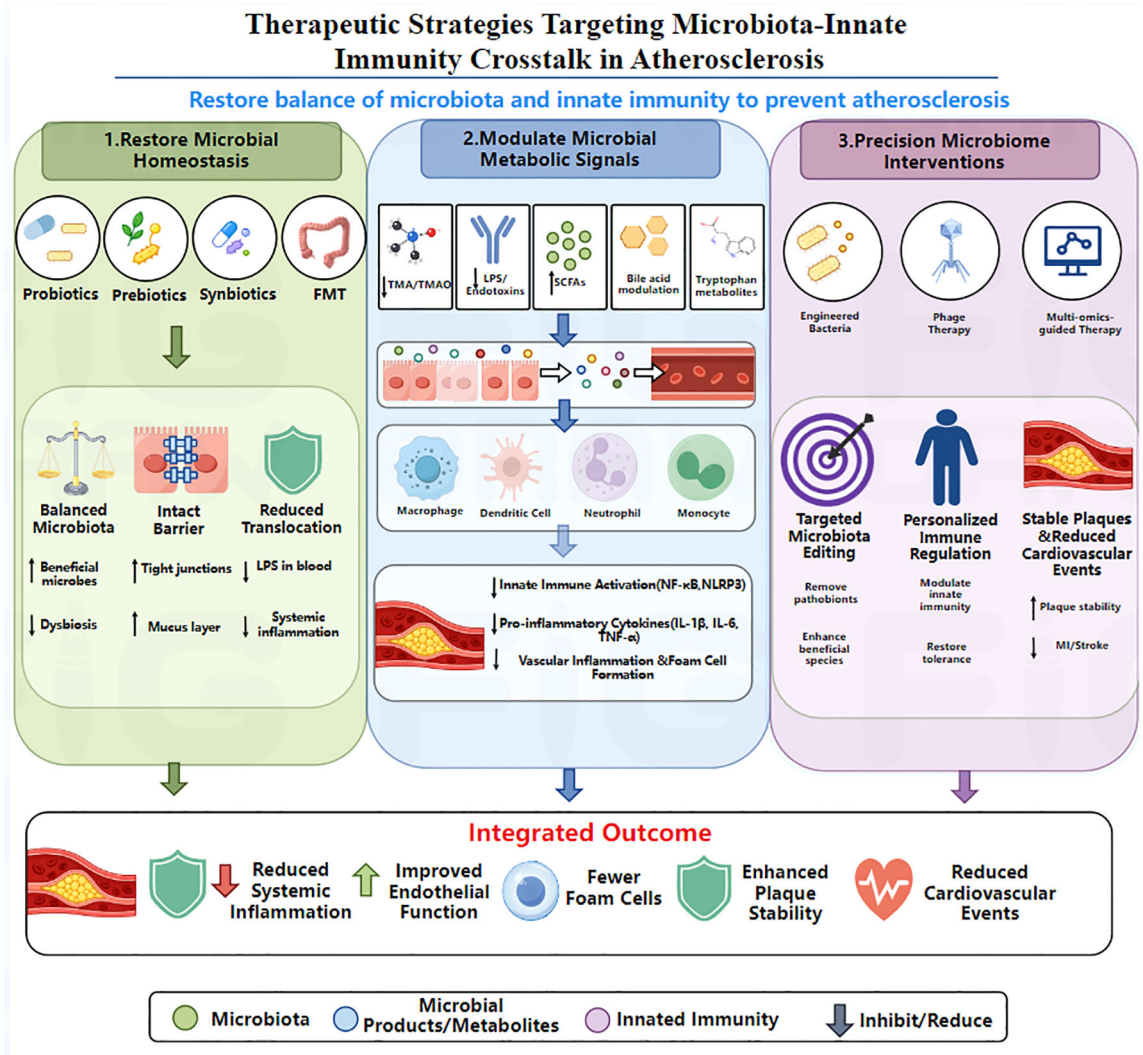
Therapeutic strategies targeting microbiota–innate immunity crosstalk in atherosclerosis. Current therapeutic approaches targeting the microbiota–innate immunity axis can be broadly categorized into microbiota restoration, modulation of microbial metabolic signals, and precision microbiome interventions. Microbiota restoration strategies, including probiotics, prebiotics, synbiotics, and FMT, aim to improve microbial balance, enhance barrier integrity, and reduce microbial translocation. Targeting microbial metabolic pathways focuses on reducing pro-atherogenic metabolites such as TMAO and endotoxins while increasing protective metabolites including SCFAs and beneficial bile acid and tryptophan-derived metabolites. Emerging precision interventions, such as engineered bacteria, phage therapy, and multi-omics-guided therapeutic approaches, enable targeted microbiota editing and personalized immune regulation. Collectively, these strategies reduce innate immune activation and vascular inflammation, improve endothelial function, decrease foam cell formation, enhance plaque stability, and ultimately reduce cardiovascular events.

With the growing recognition of the role of microbiota–innate immune crosstalk in the initiation and progression of atherosclerosis, therapeutic strategies targeting this regulatory network have emerged as an important and rapidly expanding area of research. At present, these interventions can be broadly categorized into three main approaches, including microbiota remodeling, targeted modulation of microbial metabolic pathways, and emerging precision-based therapeutic strategies (**Table 2**). Collectively, these approaches exert anti-atherosclerotic effects through multiple complementary mechanisms, as summarized in **Figure 3**.

**Table 2:** Therapeutic strategies targeting the microbiota–innate immune axis in atherosclerosis.

Intervention strategy	Mechanisms of action and atherosclerosis-related effects	Development stage	References
Probiotics	Increase beneficial microorganisms such as Lactobacillus and Bifidobacterium, promote short-chain fatty acid production, suppress Toll-like receptor 4/nuclear factor-κB signaling and NLR family pyrin domain-containing 3 inflammasome activation, and reduce vascular inflammation and foam cell formation	Preclinical studies/Clinical studies	(136)
Prebiotics	Promote the expansion of short-chain fatty acid–producing microorganisms, increase levels of metabolites such as butyrate, enhance barrier integrity, reduce lipopolysaccharide translocation into the circulation, and attenuate monocyte recruitment and pro-inflammatory macrophage activation	Preclinical studies/Clinical studies	(137)
Synbiotics	Combine probiotics and prebiotics to synergistically improve microbial ecology and metabolic function, reduce inflammatory mediator production, and ameliorate dyslipidemia and vascular inflammation	Preclinical studies/Clinical studies	(138)
Fecal microbiota transplantation (FMT)	Reconstruct microbial ecosystems, restore microbial diversity and metabolic homeostasis, reduce enrichment of pro-inflammatory microorganisms and microbial-derived danger signals, and alleviate atherosclerosis-associated inflammation	Preclinical studies/Early exploratory studies	(139)
Trimethylamine N-oxide pathway inhibitors	Inhibit the conversion of choline and carnitine into trimethylamine and trimethylamine N-oxide, reduce scavenger receptor expression, NLR family pyrin domain-containing 3 inflammasome activation, and foam cell formation, and delay plaque progression	Preclinical studies/Translational studies	(140)
Short-chain fatty acid supplementation	Modulate immunometabolism through free fatty acid receptors 2 and 3, G protein-coupled receptor 43 signaling, and histone deacetylase inhibition, thereby reducing the release of pro-inflammatory cytokines such as interleukin-1β and tumor necrosis factor-α and attenuating vascular inflammation	Preclinical studies/Mechanistic studies	(141)
Bile acid signaling modulators	Regulate cholesterol metabolism and immune responses through farnesoid X receptor and Takeda G protein-coupled receptor 5 signaling, suppress pro-inflammatory macrophage activation, improve endothelial function, and enhance plaque stability	Preclinical studies/Clinical studies	(57)
Tryptophan metabolism modulators	Regulate barrier homeostasis and immunometabolism through aryl hydrocarbon receptor and pregnane X receptor signaling, suppress aberrant inflammatory responses, and reduce endothelial injury and excessive myeloid cell activation	Mechanistic studies/Preclinical studies	(142)
Engineered microbial therapeutics	Utilize genetically engineered microorganisms to produce anti-inflammatory metabolites, degrade harmful metabolites, or deliver immunomodulatory molecules, thereby enabling precise regulation of microbiota–innate immune interaction networks	Emerging research	(143)
Bacteriophage therapy	Selectively eliminate pro-inflammatory microorganisms or trimethylamine-producing bacterial populations, reduce microbial-derived danger signals and metabolic abnormalities, restore microbial homeostasis, and attenuate vascular inflammation	Emerging research	(144)

Collectively, the microbiota–innate immune–targeted strategies discussed herein are primarily aimed at early disease prevention, while also exhibiting therapeutic potential in established disease. This continuum underscores an evolving paradigm shift from conventional risk factor modification toward precise modulation of disease-driving molecular and immunological pathways.

### 5.1 Microbiota remodeling strategies

Microbiota remodeling represents one of the most extensively investigated approaches for targeting the microbiota–innate immune axis. The primary objective of these interventions is to restore microbial ecological balance, reduce the generation of pro-inflammatory signals, and re-establish host immune homeostasis. Among currently available strategies, probiotics, prebiotics, and synbiotics have received the greatest attention.

Probiotics can ameliorate dysbiosis through multiple mechanisms, including competitive inhibition of potentially pathogenic microorganisms, enhancement of intestinal barrier integrity, and modulation of microbial metabolic activity. In addition, probiotic-derived short-chain fatty acids suppress Toll-like receptor 4/nuclear factor-κB signaling and NLR family pyrin domain-containing 3 inflammasome activation, thereby reducing the production of pro-inflammatory mediators such as interleukin-1β and tumor necrosis factor-α while promoting macrophage polarization toward anti-inflammatory phenotypes. Prebiotics, in contrast, selectively stimulate the growth and metabolic activity of beneficial microorganisms, increasing short-chain fatty acid production, strengthening barrier function, and reducing the translocation of lipopolysaccharide into the circulation. Synbiotics combine probiotics and prebiotics, providing both beneficial microbial strains and substrates that facilitate their colonization and persistence. Consequently, synbiotic interventions may exert synergistic effects on microbial community restoration, lipid metabolism regulation, and inflammatory suppression.

Fecal microbiota transplantation (FMT) represents a promising strategy for global reconstruction of intestinal microbial ecology. By transferring a healthy donor-derived microbial community, FMT can restore intestinal ecosystem structure and metabolic function, thereby partially correcting dysbiosis. However, its application in atherosclerosis remains largely at the preclinical and early exploratory stages, with limited clinical evidence and a lack of large-scale randomized controlled trials to substantiate its therapeutic efficacy. In addition, important uncertainties persist regarding long-term safety, donor selection criteria, and inter-individual variability in treatment response.

Overall, microbiota remodeling strategies hold considerable translational potential by restoring microbial homeostasis, reducing pro-inflammatory microbial signals, and modulating innate immune activity. Nevertheless, these approaches remain in the early stages of clinical validation and require further mechanistic and interventional studies to fully establish their therapeutic robustness in atherosclerosis.

### 5.2 Targeting microbial metabolic pathways

Beyond restructuring microbial communities, direct modulation of microbial metabolites and their associated immune pathways has emerged as another important therapeutic strategy in atherosclerosis research. Patients with atherosclerosis often exhibit not only accumulation of pro-inflammatory metabolites but also depletion of metabolites with protective immunoregulatory functions, resulting in persistent inflammatory activation, dysregulated lipid metabolism, and vascular dysfunction (145). Consequently, reducing harmful metabolite production, enhancing beneficial metabolic signals, and restoring metabolic–immune homeostasis may provide an effective means of interrupting disease progression driven by microbiota–innate immune interactions.

Current efforts have focused primarily on two complementary approaches: suppression of pro-atherogenic metabolites and enhancement of protective metabolic pathways. Trimethylamine N-oxide is among the most extensively studied pro-atherogenic microbial metabolites. By promoting macrophage lipid uptake, inflammasome activation, and foam cell formation, this metabolite contributes directly to lesion development and progression. Accordingly, pharmacological inhibition of trimethylamine generation or blockade of downstream signaling pathways has attracted considerable interest as a potential therapeutic strategy.

Conversely, several microbial metabolites exert protective effects on vascular and immune homeostasis. Short-chain fatty acids, selected bile acid metabolites, and tryptophan-derived compounds contribute to the maintenance of barrier integrity, regulation of immunometabolism, and suppression of inflammatory responses. Increasing the abundance or biological activity of these metabolites may attenuate macrophage-driven inflammation, improve endothelial function, and enhance plaque stability.

Multiple metabolic pathways collectively regulate host immunometabolic networks; thus, future interventions are likely to move beyond single-metabolite targeting toward integrated modulation of the microbial metabolic landscape to restore microbiota–immune–metabolic homeostasis and enable precision intervention in atherosclerosis. Moreover, commonly used atherosclerosis-related drugs, particularly statins (e.g., atorvastatin), antibiotics, and antidiabetic agents, can modulate gut microbiota composition and function and thereby indirectly influence the microbiota–innate immune axis (146).

### 5.3 Emerging precision intervention strategies

Advances in multi-omics technologies, synthetic biology, and precision medicine have accelerated the transition from broad-spectrum microbiota modulation toward more targeted therapeutic approaches aimed at the microbiota–innate immune axis. Unlike conventional interventions that broadly alter microbial communities or metabolic pathways, emerging precision strategies seek to selectively manipulate specific microorganisms, metabolic circuits, or immune responses, thereby maximizing therapeutic efficacy while minimizing unintended off-target effects.

Engineered microbial therapeutics have emerged as one of the most rapidly evolving areas of research. Through rational genetic modification, engineered bacteria can be designed to enhance the production of beneficial metabolites such as short-chain fatty acids, suppress the generation of pro-atherogenic metabolites including trimethylamine N-oxide precursors, or promote the expression of anti-inflammatory mediators. By modulating macrophage function, restoring immunometabolic balance, and attenuating vascular inflammation, these engineered microorganisms may provide therapeutic benefits beyond those achievable with conventional probiotics. Their programmable nature and potential for personalized design further highlight their promise as next-generation therapeutic platforms.

Bacteriophage-based therapy represents another attractive strategy for precise microbiome modulation. By selectively targeting specific bacterial populations, bacteriophages can eliminate pathogenic or pro-inflammatory microorganisms while preserving the overall ecological stability of the microbial community. This high degree of host specificity distinguishes bacteriophage therapy from traditional antimicrobial approaches, which often induce broad alterations in microbial composition. Recent studies have suggested that targeting trimethylamine-producing bacteria and other pro-inflammatory microbial populations may improve host metabolic profiles and reduce inflammatory burden, supporting the potential utility of bacteriophage-based interventions in atherosclerosis management.

In addition, precision microbiota-targeted interventions based on multi-omics data integration have gained increasing attention. Recent evidence suggests that gut microbiota-associated non-coding RNA regulatory networks play important roles in cardiovascular diseases (147). Meanwhile, advances in metagenomics, metabolomics, and single-cell sequencing technologies have enabled increasingly refined characterization of atherosclerosis-associated key microbial taxa, metabolic pathways, and immune dysregulation profiles. On this basis, the integration of artificial intelligence–assisted analytical approaches with individualized microbiome profiling may facilitate the development of patient-specific precision intervention strategies. This paradigm shift supports a transition from simple modulation of microbial community structure toward systematic reconstruction of the microbiota–immune regulatory network.

Importantly, the biological effects of microbiota-targeted interventions and metabolic pathway modulation are highly time-dependent. Microbial compositional changes may occur relatively rapidly, whereas immunological remodeling and clinical benefits often exhibit delayed onset and substantial inter-individual variability. Overall, although emerging precision intervention strategies remain at an exploratory stage, they hold considerable promise for improving therapeutic specificity, enhancing efficacy, and advancing personalized treatment approaches. With continued mechanistic and translational research, precise modulation of the microbiota–innate immune axis is expected to become a key direction in future atherosclerosis prevention and treatment.

## 6 Conclusions and future perspectives

Atherosclerosis is a complex multifactorial disease driven by metabolic dysregulation, chronic inflammation, and immune imbalance. Gut microbiota and its structural components, metabolites, and secreted factors regulate the functions of macrophages, neutrophils, dendritic cells, and endothelial cells through pattern-recognition receptors and immunometabolic signaling pathways. Conversely, the innate immune system shapes gut microbial composition and metabolic activity through barrier defense and inflammatory regulation, thereby establishing a dynamic and bidirectional interaction network that spans the entire course of disease progression and contributes to key pathological processes, including endothelial dysfunction, foam cell formation, and plaque progression.

Despite substantial advances in recent years, most current evidence is derived from animal models and association-based studies. The causal relationships and temporal dynamics between microbial dysbiosis and innate immune reprogramming remain incompletely defined. In addition, marked inter-individual variability in microbiota composition—shaped by genetic background, dietary patterns, medication exposure, and comorbid conditions—further complicates biomarker identification and clinical translation. Future studies should integrate multi-omics approaches with longitudinal monitoring to systematically characterize microbiota–immune interaction networks and identify key regulatory nodes. Moreover, well-designed prospective cohort studies and randomized controlled trials are needed to validate the predictive and therapeutic potential of candidate biomarkers. With the rapid development of engineered microbial therapeutics, bacteriophage-based interventions, and targeted metabolic modulation strategies, precise regulation of the microbiota–innate immune axis is expected to become a central direction in atherosclerosis prevention and treatment.

To facilitate a concise understanding of the major findings of this review, the key conclusions are summarized as follows. First, the gut microbiota contributes to atherosclerosis initiation and progression through multiple mechanisms involving metabolites, structural components, and genetic materials, playing a central role in vascular inflammatory regulation. Second, the innate immune system serves as a critical interface linking gut microbiota and vascular pathology, and its functional alterations are pivotal in disease progression. Third, microbial-derived metabolites regulate inflammatory responses and plaque stability through multiple signaling pathways, including HIF-1α, NLRP3, AhR, and FXR. Fourth, microbiota–innate immune crosstalk exhibits stage-dependent dynamic regulation and operates throughout the entire disease continuum. Finally, although microbiome-based interventions hold considerable translational promise, their safety and efficacy require further validation through high-quality clinical studies.
